# Getting under the Skin: Report from the International Psoriasis Council Workshop on the Role of Stress in Psoriasis

**DOI:** 10.3389/fpsyg.2016.00087

**Published:** 2016-02-02

**Authors:** Julia Schwartz, Andrea W. M. Evers, Christine Bundy, Alexandra B. Kimball

**Affiliations:** ^1^Department of Dermatology, George Washington UniversityWashington, DC, USA; ^2^Department of Health, Medical, and Neuropsychology, Leiden UniversityLeiden, Netherlands; ^3^Department of Medical Psychology, Radboud University Medical CenterNijmegen, Netherlands; ^4^Centre for Dermatology Research, University of Manchester and Manchester Academic Health Sciences CentreManchester, UK; ^5^Harvard Medical SchoolBoston, MA, USA

**Keywords:** psoriasis, stress, depression, anxiety, psychological comorbidities, hypothalamic-pituitary-adrenal axis, cognitive behavioral therapy

## Abstract

Psoriasis is a chronic inflammatory skin condition with significant physical and psychosocial comorbidity. A workshop of leading experts in dermatology and psychology with the purpose of better understanding the current role of psychological comorbidities in psoriasis was held by the International Psoriasis Council in November 2013. The role of stress reactivity with a focus on the hypothalamic-pituitary-adrenal axis was emphasized. While cognitive behavioral therapy remains the most extensively studied and successful treatment strategy in patients with psoriasis and various psychological comorbidities, new and innovative interventions such as online-based therapies have recently emerged. Strategies and recommendations toward approaching psychological comorbidities are discussed.

## Introduction

Psoriasis is a chronic, relapsing and remitting inflammatory disease of the skin with a prevalence of 2–3% worldwide (Koo, 1999). Chronic plaque psoriasis, the most common form of this disease, is characterized by scaly, erythematous, and infiltrated skin lesions, which are often pruritic and painful (Christophers and Mrowietz, 2003). Psoriasis can be a significantly disabling disease, interfering with activities of daily life, increasing levels of social stigmatization, and generating psychological distress. Stress is a well-known exacerbating factor of psoriasis (Farber and Nall, 1993). The interplay between stress and other psychological comorbidities with psoriasis has been of common interest to both dermatologists and mental health professionals alike.

The International Psoriasis Council (IPC) is a dermatology-led, global non-profit organization dedicated to innovation across the full spectrum of psoriasis through research, education, and treatment. Recently, the IPC has led an initiative to better define the association of various psychological comorbidities with psoriasis. In November 2013, a workshop was held in Boston, MA, which assembled a panel of global dermatology and psychology experts, with the objective to review the current research in the role of psychological comorbidities, focusing on stress and the interplay of the hypothalamic-pituitary-adrenal axis (HPAA) in psoriasis. In addition, the committee also sought to describe the current psychological treatment strategies that have been studied in psoriatic patients with recommendations for clinical practice. The following report synthesizes the presentations and panel discussions from this meeting.

## HPAA activity and stress reactivity in psoriasis

Andrea Evers discussed the connection between stress reactivity and psoriasis. While the role of stress in the escalation of skin disease and psoriasis is a common patient-reported phenomenon, there have been relatively few experimental studies on the correlation between psoriasis and stress exposure (Fortune et al., 2005). Prospective studies have shown that periods of high stress moderate the disease course in psoriasis, with more daily stressors predicting increased Psoriasis Area and Severity Index (PASI) scores with itch in the following month. (Verhoeven et al., 2009; Evers et al., 2010) One potential mediator of the stress and skin disease relationship is the role of the HPAA, which may be dysregulated in patients with chronic inflammatory diseases. Gene expression analysis studies have shown increased expression of several HPAA mediators in both lesional and non-lesional psoriasis skin samples compared to normal skin controls (Loite et al., 2013). Experimentally induced stress tests have shown that cortisol levels in patients with psoriasis exposed to a public speaking task are significantly more acutely elevated than in healthy controls (de Brouwer et al., 2013b). This phenomenon may be influenced by different patient characteristics, however, as subgroups of patients who consider themselves highly stress reactive actually have lower baseline and post stressful task cortisol levels compared to those that do not describe themselves as stress reactive. (Richards et al., 2005; Evers et al., 2010) This attenuated adrenal activity has also been observed in other chronic inflammatory diseases such as atopic dermatitis and rheumatoid arthritis, and could potentially contribute to the perpetuation of the inflammatory state in these diseases. (Chikanza et al., 1992; Buske-Kirschbaum et al., 2002) This particular subgroup of high stress responders who exhibit a hypocortisolemic response to stress are speculated to also be more likely to have more severe rebound flares of their psoriasis following discontinuation of systemic corticosteroids (Richards et al., 2005).

So is it possible to change the psychophysiological stress response in psoriasis with stress-management treatment? New research implies that stress relief interventions can lead to changes in the cortisol responses of highly distressed individuals. Stress management training has been shown to positively affect the course of psoriasis and quality of life of patients in addition to contributing to a reduction of treatment duration when used in conjunction with certain mainstay therapies for psoriasis, such as a phototherapy. (Kabat-Zinn et al., 1998; Fortune et al., 2002) Healthy subjects who underwent resource-oriented stress management training prior to an experimentally induced stress task had significantly attenuated cortisol responses and stress appraisals than controls (Storch et al., 2007). While the data for psoriasis has been modest to date, immune modulation has been observed in patients with rheumatoid arthritis who participated in stress management training, with a significant reduction of Il-8 cytokine levels at 2 month follow-up compared to a control (de Brouwer et al., 2013a). Therapies targeting stress management have therefore been a focus of adjunct treatment in these patients in an effort to reduce flares.

Studies have shown that there is a lack of effective systematic screening for subgroups at risk (e.g., highly distressed patients). A questionnaire study of dermatologists in The Netherlands demonstrated that on average, dermatologists referred only eight patients per year to a psychologist or other psychosocial professional and 50% of dermatologists have never referred to a psychologist or other psychosocial therapist (Evers et al., 2013). Countries, such as the Netherlands, have national referral networks for psychosocial professionals with educational programs and resources for referring providers. Examples of tailored care approaches to stress-management were discussed. One such approach is the use of E-health applications, which are web-based programs that use online screening tools to identify sub-groups within a population and then offer patient-specific tailored training based on those subgroups. This method may offer greater patient flexibility and financial and time economization when access to a face-to-face specialized cognitive behavioral therapist is limited. In target groups, such as those with relatively severe adjustment problems who may benefit from this type of intervention, web-based therapies have shown to be effective in various psychological conditions including anxiety and depression in addition to chronic somatic conditions (Cuijpers et al., 2008). The implications for clinical practice are thus, to reduce stress in these patients through the establishment of trust in care, effective doctor-patient communication, and providing adequate information of risk and treatment options.

## Psychological intervention in psoriasis

Elaborating further on treatment in the realm of psychological comorbidities, Christine Bundy reviewed the currently available psychological interventions for people with psoriasis. While a range of psychological interventions have been used in this population, there have been few high-quality studies comparing their effectiveness in this population. Cognitive behavioral therapy (CBT), including one-on-one, group-based, and online programs, is probably the most widely studied treatment with clear evidence that an emotion-focused CBT approach can have a positive effect on psoriasis activity, distress, and quality of life, especially if therapy is tailored to the individual. (Zachariae et al., 1996; Fortune et al., 2002, 2004; Bundy et al., 2013) Other cognitive-based approaches including education and self-management, mindfulness, and psychotherapy have limited supporting data due to poor quality studies. There is almost no evidence to support claims that a purely educational approach to patient management improves psychological outcomes. A study by Bostoen and colleagues did show a relatively small effect on PASI, DLQI, depression, physical activity, and smoking cessation for a combined education and self-management approach, however, inference is limited due to a small sample size and a relatively unrealistic management strategy using a multi-professional team of nine people for 2 h twice per week for 3 months (Bostoen et al., 2012). Similarly, studies in arousal management and mindfulness training are lacking in high-quality reporting and therefore, firm conclusions on their effectiveness in treating patients with psoriasis with psychological comorbidities cannot be confidently stated (Fordham et al., 2013).

The importance of recognizing increased comorbidities such as obesity, smoking and alcohol abuse in patients with psoriasis were highlighted together with strategies in behavior change therapies. Motivational interviewing including self-management does effect behavior change in patients with other long-term conditions such as cardiovascular and diabetes (Knight et al., 2006; Phillips et al., 2012). Studies in weight reduction using a very low energy diet showed significant weight reduction as well as trends in PASI improvement and significant reductions in DLQI in overweight patients with psoriasis (Jensen et al., 2013). Unfortunately, there are no reliable studies in alcohol reduction and smoking cessation in this population.

In summary, a tailored CBT approach has been most consistently effective in managing distress in patients with psoriasis. However, CBT has high rates of attrition even with online delivery and the clinical dermatology workforce is not skilled to deliver CBT interventions with too few psychologists available. A combination strategy of both CBT and behavior change therapy has potential to optimally treat this population. More high quality trials with larger samples and consistent outcome measures on behavior change interventions focusing on weight, physical activity, alcohol use, and smoking are needed in order to manage cardiovascular disease and diabetes risk. Accessible interventions targeting both psychological and physical outcomes that work in a standard clinic setting are needed. Dermatologists need to be familiar with all available therapeutic options and refer appropriately. Lastly, staff training to support specialist interventions is recommended with the emphasis on demonstrating the fidelity of interventions and consistency of approach among staff.

In conclusion, psoriasis is a chronic disease that can carry a significant psychological comorbidity burden. Increased stress has been shown to have a negative impact on skin involvement, further potentiating the disease process. New research suggests that this clinical response may be related to cortisol dysregulation within the HPAA and that the psychophysiological stress response in patients with psoriasis may be improved with stress-management treatment. E-health therapy, among other approaches, for example, may be a flexible, patient-tailored approach to stress reduction in this population. A multidisciplinary approach utilizing both dermatologic care with screening of comorbidities and early referral access to psychological intervention may be crucial and should be encouraged toward optimal management of this disease.

## Author contributions

JS led the development of the paper and provided content and edits. AE provided content on psoriasis for the development of this manuscript. CB provided content to the development of the manuscript in terms of pycho-social issues in psoriasis. AK led the workshop and development of the paper.

## Funding

International Psoriasis Council (IPC) is a non-profit charity organization that received unrestricted sponsorship from Abbvie & Amgen to support the workshop. The sponsors had no influence on the content of the program or the viewpoints in this manuscript.

### Conflict of interest statement

Alexandra B. Kimball is an Investigator and Consultant and receives grants and honoraria from Abbott, Centocor and Amgen. Julia Schwartz has partial fellowship funding from Janssen.
